# NMR‐based metabolomic investigation of dogs with acute flaccid paralysis due to tick paralysis

**DOI:** 10.1002/vms3.1528

**Published:** 2024-07-02

**Authors:** Erdem Gülersoy, Canberk Balıkçı, Adem Şahan, İsmail Günal, Mehmet Osman Atlı

**Affiliations:** ^1^ Veterinary Faculty Department of Internal Medicine Harran University Şanlıurfa Turkey; ^2^ Veterinary Faculty Department of Reproduction and Artificial Insemination Harran University Şanlıurfa Turkey

**Keywords:** acute flaccid paralysis, dog, nuclear magnetic resonance, tick

## Abstract

**Background:**

Acute flaccid paralysis (AFP) is a complex clinical syndrome with various aetiologies. If untreated, AFP may lead to death due to failure of respiratory muscles. Tick paralysis, which is a noninfectious neurologic syndrome of AFP, occurs following tick attachment, engorgement, and injection of tick saliva toxins. There is no specific diagnostic test for tick paralysis, and mortality increases as definitive diagnosis is delayed. Although metabolomic investigation of tick saliva was conducted, there is a lack of research on metabolomic evaluation of hosts affected by tick paralysis.

**Objectives:**

Thus, the aim of this study is to investigate metabolomic changes in serum samples of dogs with tick paralysis due to *Rhipicephalus sanguineus* using NMR‐based metabolomics and to identify potential diagnostic/prognostic markers.

**Materials and Methods:**

Forty dogs infested with *R. sanguineus*, with clinical findings compatible with AFP and with a confirmed tick paralysis diagnosis ex juvantibus, constituted the Paralysis Group. Ten healthy dogs, which were admitted either for vaccination and/or check‐up purposes, constituted the Control Group. After the confirmation tick paralysis, medical history, vaccination and nutritional status, body surface area and estimated tick numbers of all the dogs were noted. Physical examination included body temperature, heart and respiratory rate, capillary refill time evaluation and Modified Glasgow Coma Scale calculation. Serum samples were extracted from venous blood samples of all the dogs and were prepared for NMR analysis, and NMR‐based metabolomics identification and quantification were performed.

**Results:**

NMR‐based serum metabolomics of the present study revealed distinct up/down‐regulated expressions, presenting a promising avenue. Moreover, it was observed that energy metabolism and especially liver functions were impaired in dogs with tick paralysis, and not only the respiratory system but also the kidneys were affected.

**Conclusion:**

It was concluded that the present approach may help to better understand the pathological mechanisms developing in cases of AFP due to tick paralysis.

## Graphical abstract

The NMR‐based serum metabolomics approach used in this study revealed distinct up‐regulated/down‐regulated expressions, presenting a promising avenue for research. It was observed that energy metabolism, particularly liver function, was impaired in dogs with tick paralysis. Additionally, not only the respiratory system but also the kidneys were affected. This approach may help to better understand the pathological mechanisms developing in cases of tick paralysis. Furthermore, the detected markers can be used for diagnostic and prognostic purposes.

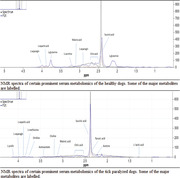

## 1 INTRODUCTION

Acute flaccid paralysis (AFP) manifests as a clinical syndrome characterized by the rapid onset of weakness, occasionally involving the respiratory and swallowing muscles. The term flaccid denotes the absence of spasticity or other indicators of disordered central nervous system motor tracts, such as clonus, hyperreflexia or extensor plantar responses (Marx et al., 2000). AFP reaches its maximum severity within a span of days to weeks, with the progression timeline being contingent upon the specific aetiology (Bowley & Chad, 2019). Aetiologies contributing to AFP encompass spinal cord injuries (infectious, inflammatory and compressive), neuromuscular junction disorders (myasthenia gravis, botulism and Eaton‐Lambert Myasthenic Syndrome), muscle‐related issues (muscular dystrophies, necrotizing myopathies, hypokalemia or severe hyperkalemia) and root or peripheral nerve disorders (Guillain–Barré syndrome, polyneuropathy and tick paralysis) (Fadia et al., 2019).

Certain tick species, such as *Ixodes holocyclus*, can inoculate neurotoxins (secreted by their salivary glands during blood feeding) that induce a rapid, ascending flaccid paralysis in animals (Otranto et al., 2012). Tick paralysis is an acute, progressive ascending motor paralysis caused by a salivary neurotoxin produced by ticks (Mans et al., 2004). The neurotoxins could disrupt the release of acetylcholine at the neuromuscular junction, leading to the generation of a neuromuscular blockade (Edlow & McGillicuddy, 2008). Cases of paralysis by ixodid ticks have been reported in several animal species, including snakes, birds, cattle, cats and humans. In spite of the broad distribution of *Rhipicephalus sanguineus* (*R. sanguineus*) and previous reports (Gülersoy & Günal, 2022), the role of this tick species as cause of flaccid paralysis in dogs remains anecdotic (Otranto et al., 2012).

Clinical medicine encompasses disease prevention, diagnosis and treatment, with biomarkers playing a pivotal role in each of these domains. Omics technologies, including genomics, transcriptomics, proteomics, and metabolomics, provide valuable tools for the discovery of biomarkers. Since the start of the 21st century, metabolomic technologies have furthered the understanding of certain molecular mechanisms that underlie diseases (Chen et al., 2011). Early diagnosis of tick paralysis, which is one of the causes of AFP, is vital because of its highly variable prognosis. Treatment in confirmed cases primarily involves supportive measures, with the exception of tick‐antitoxin serum application. Identifying metabolites with altered concentrations in dogs affected by tick paralysis may offer novel markers for the diagnosis and treatment of the disease. While vaccinomics, an approach leveraging omics technologies and bioinformatics, has been explored in the context of tick vaccination (de la Fuente & Merino, 2013), and the responses of ticks to certain pesticide applications have been scrutinized using metabolomic approaches, there has been a notable absence of research into the metabolomic evaluation of hosts affected by tick paralysis (Pathak et al., 2022). Therefore, the aim of this study is to investigate metabolic changes in serum samples of dogs with tick paralysis due to *R. sanguineus* infestation using NMR‐based metabolomics and to identify potential markers with diagnostic and prognostic value in the diagnosis and treatment of the disease.

## 2 MATERIALS AND METHODS

### 2.1 Animals

From April to October 2022, 40 dogs were referred to the teaching hospital of the Faculty of Veterinary Medicine (Harran University, Şanlıurfa, Türkiye) with sudden neurological disorders such as weakness, difficulty in movements, hind limb incoordination and quadriplegia. Dogs were client‐owned and of mixed‐breed, different sex, aged 4–6 months. Medical history, vaccination and nutritional status, body surface area (BSA) and estimated tick numbers of all the dogs were noted.

### 2.2 Physical examinations

Criteria for inclusion in the study were that the dog had no history of any disease, had not been administered antiparasitic medication recently (<1 month), had an engorged tick on it, and had signs of AFP. The present study classifies the following clinical findings as suggestive of AFP: an inability to contract due to motor pathway impairment extending from the cortex to muscle fibres; the absence of spasticity or other signs of disordered central nervous system motor tracts, such as hyperreflexia, clonus or extensor plantar responses; and the sudden development and worsening of weakness within a few days, particularly affecting respiratory muscles and swallowing ability (Growdon & Fink, 1994; Marx et al., 2000).

Within the scope of the physical examination, body temperature, heart and respiratory rate and gingival capillary refill time were evaluated and Modified Glasgow Coma Scale (MGCS) scores of all the dogs were calculated. Additionally, the body weight and BSA of each dog were calculated. Tick paralyzed dogs were evaluated for the presence of ticks by thumb counting of anatomical body regions including head, neck, ears, thorax, abdomen, interdigital areas, fore and hind limbs, tail, axillary and inguinal area. In order not to affect the NMR‐based serum metabolomic analysis results, complete blood count (CBC) and microscopic blood smear examinations were performed on all dogs included in the study (blood and buffy coat smears were examined for Anaplasma platys, Ehrlichia canis, Babesia spp. and Hepatozoon canis inclusions). Each smear was optimally prepared within 1 h of collection to ensure the best morphology and then examined for approximately 10 min for wider view under a 100× oil immersion objective using a light microscope. As a result of clinical examinations, dogs with any blood parasites and findings such as thrombocytopenia and pancytopenia, which have been commonly reported in dogs previously infected with *R. sanguineus* (Otranto et al., 2012), were not included in the study.

### 2.3 Inclusion/exclusion criteria

The primary differential diagnosis for the observed clinical presentation in affected dogs encompasses other potential causes of diffuse lower motor neuron diseases, such as botulism, acute idiopathic polyneuropathy and snake envenomation (Malik & Farrow, 1991). In brief, botulism in dogs typically arises concurrently with the ingestion of spoiled food or carrion, a scenario not applicable to the dogs under consideration as they were exclusively fed commercial dry dog food. Clinically, botulism manifests as challenges in food prehension and swallowing, accompanied by saliva drooling and regurgitation (Shelton, 2002). Dogs exposed to raccoon saliva or those with a history of prior systemic disease have been reported to develop acute idiopathic polyneuropathy, characterized by hyperesthesia and neurogenic muscular atrophy persisting for more than five to 7 days (Malik & Farrow, 1991). Although acute idiopathic polyradiculoneuritis has been known to involve the cranial nerves and induce respiratory paresis or paralysis due to involvement of the intercostal or phrenic nerves, most occurrences of clinical symptoms are restricted to the limbs (Martinez‐Anton et al., 2018). In the mentioned neuromuscular paralysis conditions, the respiratory pattern is typically rapid and shallow. However, in the present cases, it was slow and marked by an expiratory effort, resembling the pattern observed in tick paralysis (Holland, 2008). Myopathy was also ruled out since it is characterized by proximal weakness or fatigue, normal sensitivity and subsequent loss of reflexes, typically after significant atrophy (Cuddon, 1998). Thus, although the neurological manifestations described here are similar to other common potential findings of lower motor neuron diseases resembling tick paralysis (Musteata et al., 2020), the cases were confirmed by the rapid improvement observed after acaricidal treatment and tick removal (Malik & Farrow, 1991).

Dogs with findings indicating the presence of AFP but no ticks could be detected on them, dogs with a different aetiology of AFP as a result of anamnesis, clinical and laboratory examinations, and dogs with other aetiologies causing neurological disorders such as spinal cord pressure, epidural abscesses and plant and snake toxins were not included in the study. Confirmation of tick paralysis caused by *R. sanguineus* was achieved through the ex juvantibus method, as all dogs showed prompt and full recovery (median: 24 h, min: 16 h, max: 34 h) after acaricidal treatment and tick removal. The collected ticks were stored individually in vials and later identified at the species level using morphological keys. As a result, 40 dogs infested with *R. sanguineus*, with clinical findings compatible with tick paralysis and with a confirmed tick paralysis diagnosis ex juvantibus, constituted the Paralysis Group of the study. Ten dogs with similar BSAs (*p *< 0.796) and similar ages (*p* < 0.513), which were admitted either for vaccination and/or check‐up purposes, constituted the healthy Control Group.

### 2.4 Taking venous blood samples

Venous blood samples were taken from all dogs via vena cephalica (5–10 mL) venepuncture with minimal restraint in order to not cause stress. Tubes without anticoagulant were used for serum extraction (1000× rpm for 15 min at 4°C). The samples were stored in Eppendorf type tubes and kept at −80°C until the analysis.

### 2.5 Preparation of samples and NMR analysis

Before the metabolomic experiment, the serum samples underwent a thawing process at room temperature, followed by vortexing. Subsequently, 200 μL of serum was combined with 400 μL of a saline solution (0.9% NaCl, 15% deuterated water (D_2_O), containing 3 mM 3‐(trimethylsilyl)‐[2,2,3,3‐d4]‐1‐propionate [TSP]). After centrifugation (12,000× rpm, 10 min), 550 μL of the supernatant was transferred to a 5‐mm NMR tube, and the samples were stored at 4°C until measurement.

Before NMR analysis, the serum samples were allowed to equilibrate to room temperature (typically for 30 min) and then centrifuged at 7000× rpm for 10 min. Sample preparation involved transferring a 540 μL aliquot of serum to a 1.5 mL Eppendorf tube, followed by adding 60 μL of phosphate buffer solution (pH 7.5) in D_2_O. The buffer solution included 5 mM sodium TSP (used as an internal standard of known concentration) and sodium azide (used as a preservative). The final sample pH was adjusted to 7.0. The entire sample volume (600 μL) was transferred into a 5 mm NMR tube for subsequent analysis.

The ^1^H NMR spectra were obtained at 26.5°C using an Agilent 400 MHz spectrometer operating at 400.13 MHz, equipped with a 5 mm inverse detection probe featuring gradients on the *z*‐axis. Samples were run in 5 mm Wilmad 507 NMR tubes. The spectra were recorded utilizing the Nuclear Overhauser Effect Spectroscopy presaturation pulse sequence with parameters set at 32 scans, a 30 s relaxation delay, 4 s acquisition time, 8223 Hz spectral window, and collection of 64 K data points, offering a digital resolution of 0.12 Hz. Post‐acquisition free induction decay (FID) processing involved an exponential line broadening factor of 0.3 Hz. Chemical shifts are reported as *δ* values (ppm), referenced to TSP (0.0 ppm) as an internal standard. The Agilent SpectrAA software was employed for acquisition and processing. The NMR protocol allowed for full signal relaxation over a 37 s overall relaxation delay, consistent with previous works on other biological samples (Basoglu et al., 2020). Therefore, utilizing a 0.3 Hz line broadening and 37 s relaxation delay enables the use of signal intensities for quantitation purposes. In crowded NMR spectra, employing signal intensities, rather than integrals, reduces errors induced by partial signal superposition. With the collected serum kept to a minimum necessary volume, a single NMR analysis was performed with 600 μL of each sample (the regular 5 mm NMR filling volume). Opting for one measurement with the regular filling volume, as opposed to two measurements with half the filling volume, yields superior results due to increased sensitivity (higher signal‐to‐noise ratio) and improved shimming (enhanced magnetic field homogeneity leading to sharper lines in the spectrum). To minimize operator error, a single expert operator handled all NMR sample preparations and experiments. Analytical error, checked for the same operator, was well below biological variation. For four different NMR samples prepared from the same original batch, analytical error for various metabolites ranged between 1% and 5%, whereas biological variation (expressed as % RSD) fell within the range of 10%–45% (Basoglu et al., 2020; Musteata et al., 2013).

### 2.6 Identification and quantification of serum metabolomics

The identification and quantification of serum metabolomics, derived from raw 1D NMR spectra‐FID files obtained using the Agilent device, were accomplished employing the BAYESIL software. BAYESIL conducts fully automated spectral processing and spectral profiling for 1D ^1^H NMR spectra collected on standard instruments at various frequencies. In the process of spectral deconvolution, BAYESIL partitions the spectrum into small blocks, depicting sparse dependencies between these blocks through a probabilistic graphical model. Subsequently, it engages in approximate inference over this model as a surrogate for spectral profiling, yielding the most probable metabolic profile. BAYESIL, utilized for identification and quantification, encompasses a spectrum of spectral processing functions. Starting from the raw spectrum, it executes operations such as zero‐filling, Fourier and Hilbert transformations, phasing, baseline correction, smoothing, chemical shift referencing and reference deconvolution. The quantified metabolites were computed as relative concentrations of the total area. Statistical calculations for the relative concentrations of metabolites were performed using BAYESIL as well (Ravanbakhsh et al., 2015). NMR spectra visualization, simulation and presentation were performed using a software package (MestReNova, MestreLab Research).

### 2.7 Statistical analyses

Data were processed using Statistical Package for the Social Science 25.0 for Windows. The obtained values are presented as means with standard error of the mean. Distributions were analysed for each metabolites with Shapiro–Wilk test, and *p* level of less than 0.05 was regarded as significant.

## 3 RESULTS

### 3.1 Animals

In the anamnesis, it was learned that the owners collected many ticks from the dogs’ bodies before the development of paralysis and prior to hospital admission. Additionally, the anamnestic data revealed that all the dogs were unvaccinated, fed on commercial dry dog food, and lived in an outdoor kennel. At the clinical examination, additional ticks were removed from the dogs, then all were treated with a spot‐on formulation of fipronil 10%/(S)‐methoprene 9% (Frontline Combo, Merial S.A.S.).

### 3.2 Clinical examination findings

The anamnestic data of the dogs with tick paralysis revealed that the median value of symptom duration was 4 (range: 2–6) days. The median number of ticks observed in paralyzed dogs through meticulous inspection was 45 (range: 8–110). Rectal body temperature and heart rate of the Paralysis Group dogs were higher than that of the Healthy Group dogs (*p* < 0.000). The MGCS score was higher in the Healthy group than in the Paralysis Group (*p* < 0.000). Gingival capillary refill time was shorter in the paralyzed dogs than in the healthy dogs (*p* < 0.008). In the comparison of the two groups, body weight, respiratory rate, BSA and age values were statistically insignificant (*p* > 0.05). All clinical examination findings are presented in Table 1.

**TABLE 1:** Clinical examination findings.

Parameters	Paralysis Group *n* 40 mean ± SEM	Healthy Group *n* 10 mean ± SEM	*p* Value
Rectal body temp (°C)	39.54 ± 0.52	38.1 ± 0.3	0.000
Heart rate (beats/min)	108.33 ± 17.2	79.5 ± 8.68	0.000
Respiratory rate (breaths/min)	83.5 ± 9.2	79.5 ± 8.6	0.281
CRT (s)	1.8 ± 0.7	2.6 ± 0.5	0.008
MGCS	6.8 ± 3.12	16.8 ± 1.13	0.000
Body weight (kg)	5.86 ± 1.24	6 ± 1.24	0.736
BSA (m^2^)	0.32 ± 0.04	0.33 ± 0.04	0.796
Age (month)	5.06** ± **0.88	5.3** ± **0.82	0.513

Abbreviations: BSA, body surface area; CRT, capillar refill time; MGCS, Modified Glasgow Coma Scale; SEM, standard error of mean; Temp, temperature.

### 3.3 NMR‐based serum metabolomics analysis results

**FIGURE 1:**
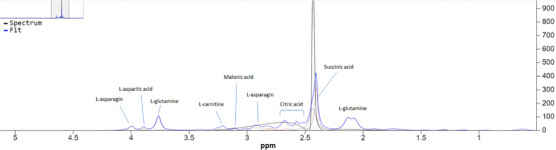
NMR spectra of certain prominent serum metabolomics of the healthy dogs. Some of the major metabolites are labelled.

**FIGURE 2:**
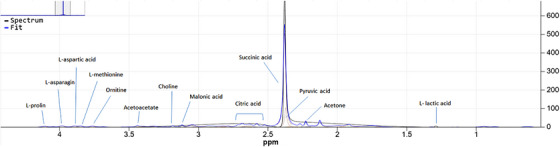
NMR spectra of certain prominent serum metabolomics of the tick paralyzed dogs. Some of the major metabolites are labelled.

Serum concentrations of betaine and l‐tryptophan were higher in the healthy dogs compared to the paralyzed dogs (*p* < 0.05). Serum acetoacetate, l‐carnitine, creatine, citric acid, choline, ethanol, d‐glucose, glycerol, formic acid, l‐glutamic acid, hypoxanthine, l‐alanine, l‐threonine, l‐asparagine, l‐isoleucine, l‐histidine, l‐serine, l‐lactic acid, pyruvic acid, urea, l‐methionine, isopropanol and acetone concentrations were higher in the paralyzed dogs than in the healthy dogs (*p* < 0.05). NMR‐based metabolomic analysis results of serum samples are presented in Table 2. NMR spectra of certain prominent serum metabolomics are presented in Figures 1 and 2.

**TABLE 2:** NMR‐based serum metabolomic analysis results.

Parameters^a^	Paralysis Group *n* 40 mean ± SEM	Healthy Group *n* 10 mean ± SEM	*p* Value
1‐Methylhistidine	47.804** ± **13.502	36.380** ± **23.017	0.140
2‐Hydroxybutyrate	278.908 ± 78.231	300.941 ± 18.021	0.155
Acetic acid	31.296 ± 20.441	37.147 ± 26.210	0.796
Betaine	76.906 ± 20.415	82.694 ± 51.339	0.015
Acetoacetate	106.866 ± 33.883	76.683 ± 61.125	0.014
l‐Carnitine	67.171 ± 17.348	10.499±5.467	0.018
Creatine	260.769 ± 126.159	15.601 ± 9.995	0.001
Citric acid	829.796 ± 38.657	584.923 ± 36.314	0.005
Choline	120.756 ± 45.148	104.417 ± 68.973	0.048
Ethanol	267.458 ± 75.266	7.116 ± 4.732	0.001
d‐Glucose	9.697 ± 6.087	1.067 ± 0.704	0.007
Glycine	88.173 ± 26.081	77.387 ± 15.688	0.318
Glycerol	40.986 ± 10.832	8.542 ± 4.909	0.023
Formic acid	59.84 ± 44.23	7.58 ± 6.88	0.000
l‐Glutamic acid	656.882 ± 43.327	98.077 ± 72.949	0.001
Hypoxanthine	64.52 ± 38.44	3.86 ± 2.1	0.022
l‐Tyrosine	456.2 ± 223.91	344.93 ± 141.27	0.372
l‐Phenylalanine	133.81 ± 44.34	75.57 ± 35.38	0.393
l‐Alanine	159.682 ± 57.140	89.639 ± 8.491	0.007
l‐Proline	123.709 ± 33.054	76.038 ± 59.191	0.128
l‐Threonine	164.595 ± 56.227	17.570 ± 11.164	0.012
l‐Asparagine	107.512 ± 39.824	57.267 ± 56.071	0.031
l‐Isoleucine	73.796 ± 26.809	2.436 ± 2.208	0.007
l‐Histidine	1.457 ± 0.628	0.302 ± 0.191	0.030
Lysine	78.234 ± 37.915	61.662 ± 19.654	0.092
l‐Serine	47.070 ± 12.297	12.833 ± 7.587	0.010
l‐Lactic acid	49.946 ± 24.280	7.044 ± 4.994	0.019
l‐Aspartic acid	146.624 ± 67.237	91.802 ± 78.965	0.218
Ornithine	79.519 ± 19.394	66.669 ± 46.217	0.151
Pyruvic acid	881.815 ± 47.226	103.990 ± 69.921	0.003
Succinic acid	869.619 ± 316.252	723.026 ± 382.651	0.411
Urea	544.132 ± 268.225	129.256 ± 54.961	0.025
3‐Hydroxybutyric acid	37.009 ± 22.139	25.892 ± 7.101	0.108
l‐Arginine	35.835 ± 18.762	25.394 ± 8.952	0.072
Creatinine	206.454 ± 73.156	203.890 ± 101.903	0.690
l‐Glutamine	201.214 ± 53.018	175.045 ± 59.109	0.206
l‐Leucine	25.805 ± 10.574	14.449 ± 6.691	0.287
Malonic acid	224.143 ± 58.426	253.397 ± 41.635	0.108
l‐Methionine	406.495 ± 233.660	51.637 ± 30.188	0.005
Isopropanol	19.046 ± 9.414	8.443 ± 1.259	0.014
l‐Valine	17.182 ± 6.235	9.026 ± 3.331	0.080
l‐Tryptophan	15.54 ± 8.77	118.92 ± 38.49	0.029
Acetone	78.305 ± 33.324	9.045 ± 8.604	0.050
Isobutyric acid	12.122 ± 4.115	8.580 ± 4.396	0.110
Methanol	59.750 ± 24.418	58.637 ± 34.562	0.818
Propylene glycol	4.496 ± 2.299	6.098 ± 3.047	0.858
Dimethylsulfone	16.714 ± 5.193	14.592 ± 11.360	0.149

Abbreviation: SEM, standard error of mean.

^a^ Concentrations in arbitrary units (mean ± SEM).

## 4 DISCUSSION

Establishing a definitive diagnosis of tick paralysis, which can cause death due to respiratory failure if untreated, not only leads to appropriate treatment but also significantly reduces the need for expensive, invasive, painful and potentially dangerous diagnostic tests and treatments (Edlow & McGillicuddy, 2008). To expedite the treatment period and enhance diagnostic capabilities, the present study used NMR‐based serum metabolomics to reveal distinct up‐ and down‐regulated expressions related to impaired liver and kidney functions, as well as energy homeostasis, presenting a promising avenue. This approach may help to better understand the pathological mechanisms developing in tick paralysis. Additionally, detected metabolites with altered expression can be used for diagnostic and/or prognostic purposes.

Metabolomics represents a novel biological analysis method aimed at delivering precise insights into small molecules (<1500 Da) associated with human metabolism. Widely employed in the realm of systems biology, metabolomics stands out for its ability to discern the most immediate metabolic changes in the body under specific conditions in a relatively short timeframe. In contrast to DNA, RNA or proteins, including enzymes, these small molecules offer an accurate reflection of such metabolic alterations. This characteristic positions metabolomics as a promising avenue for advancing clinical disease research (Wishart, 2005; Wishart, 2019). Moreover, it serves as a valuable complement to transcriptomics, genomics and proteomics, owing to its capacity to scrutinize changes in small metabolites‐distinct compounds from DNA, RNA and proteins. In comparison to other omics, metabolomics provides more direct and comprehensive information about biological systems at the individual or group level. This wealth of information lends itself to diverse applications, including disease diagnosis and treatment, analysis of drug toxicological mechanisms, the pursuit of precision medicine and among other areas (Stringer et al., 2016). In addition to these strengths, NMR is particularly suitable for detecting and characterizing sugars, organic acids, alcohols, polyols and other highly polar compounds (Emwas et al., 2019). A comprehensive request for laboratory investigations requires an understanding of both the pathology of a disease and the technical nuances of laboratory analysis. The concentrations of biological indicators in individuals with diseases can fluctuate to varying degrees over a specific time period. Although a laboratory indicator may highlight an abnormality, it may lack specificity for diagnosing a particular disease. Consequently, certain tests are employed as surrogate assessments to rule out specific diseases (El Nageh et al., 2002). When dealing with tick paralysis cases, achieving an accurate diagnosis relies on a systematic sequence of diagnostic tests. The medical history of an individual contributes vital information, including signalment, presenting clinical signs, background and details regarding the time of onset and temporal progression of clinical manifestations. Routine tests, such as a CBC, serum chemistry (including creatine kinase concentration and electrolyte measurements) and urinalysis, are conducted to establish a baseline health profile and uncover potential systemic abnormalities (Coates & Wininger, 2010; Gülersoy & Günal, 2022). NMR‐based methodologies, encompassing both targeted and untargeted metabolomics approaches, offer a valuable means to explore and comprehend the interaction between pathogens and hosts within a defined clinical context. Furthermore, specific disease states may exhibit characteristic biomarker signatures detectable through metabolomics. Consequently, validated biomarkers hold the potential for applications in disease diagnosis, prognosis, staging and the evaluation of new preclinical and clinical therapeutic agents (Tounta et al., 2021).

The protein–carbohydrates interactions are very important in immunology, biosynthesis and medicine. Animals subjected to prolonged starvation die after depletion of body proteins and death may be independent of the availability of fat for oxidative metabolism (Cherel et al., 1992). This implies that sustaining energy requirements for essential functions relies on substrates beyond acetate derived from fatty acid oxidation. The constant withdrawal or loss of intermediate metabolites from the citric acid cycle through cataplerotic reactions necessitates continuous replenishment through anaplerotic reactions. In cases of prolonged, complete starvation, amino acids may serve as the primary substrates for replenishing the 4‐carbon intermediates of the citric acid cycle and providing gluconeogenic substrates. Starvation‐induced hyperketonemia is characterized by significant increases in the quantities of ketone bodies undergoing glomerular filtration (Sapir et al., 1972). A substantial portion of acetoacetate and β‐hydroxybutyrate evades renal tubular reabsorption, leading to increased ketonuria. Although the central nervous system (brain) and other organ systems containing mitochondria may have minimal daily glucose requirements, the precise threshold has not been conclusively determined. It is widely acknowledged that during starvation, amino acids, glycerol and acetone are sourced from peripheral tissue stores to support hepatic and renal gluconeogenesis, ensuring the provision of glucose for catabolism into carbon dioxide and water (Owen et al., 1967). In previous studies, it was reported that during prolonged starvation, the brain extracts significant quantities of acetoacetate and β‐hydroxybutyrate from the blood, thus sparing the metabolism of glucose. This results in a rise in acetoacetate and β‐hydroxybutyrate (Owen, 2005). Considering the inability to feed and/or starvation of dogs with tick paralysis, high l‐glutamic acid levels can be considered a response to keep glucose levels under control to protect the function of the central nervous system, immune system or kidneys (Newsholme et al., 2003). In addition, high serum acetoacetate concentrations of the paralyzed dogs may be associated with prolonged fasting. This may be due to altered enzyme systems in liver mitochondria (Ballard et al., 1961).

Isopropanol is slowly metabolized in humans by alcohol dehydrogenase to acetone and then to acetate, formate and carbon dioxide. Acetone is metabolized by humans to acetate and formate, and reduction to isopropanol is considered a minor pathway. It was reported that serum concentrations of isopropanol and acetone were elevated in five acetonemic patients with Type I diabetes mellitus (Bailey, 1990). Acetone can be produced through endogenous metabolic pathways, particularly in lipid metabolism during conditions such as diabetic ketoacidosis or starvation. Although acetone may play a role in clinical toxicity, it is generally understood that the majority of symptoms stem from isopropanol toxicity. These symptoms encompass central nervous system depression, hypotension, hypothermia, and reduced respiratory drive (Slaughter et al., 2014). The gag reflex, also known as the pharyngeal reflex, is an involuntary reflex involving bilateral pharyngeal muscle contraction and elevation of the soft palate. In certain instances, a lack of a gag reflex may be a symptom of a more severe medical condition such as cranial nerve damage or brain death (Park et al., 2020). Depression of the gag reflex and accumulation of secretions have been reported in cases of tick paralysis, which hinders feeding (Gülersoy & Günal, 2022). The elevated serum acetone concentration observed in the tick‐paralyzed dogs in this study could be attributed to an up‐regulation in isopropanol metabolism, which may enhance the efficiency of metabolizing and eliminating isopropanol, often as a protective mechanism. Therefore, increased isopropanol concentrations may be related to a response to the inability to feed and/or starvation of the paralyzed dogs.

As the ethanol concentration in blood increases it enters all tissues and is oxidized, mostly through alcohol dehydrogenase 1 in the liver (Bach et al., 2017). Acetate, the main product of liver‐mediated ethanol oxidation, is a short‐chain fatty acid, and the oxidation of short‐chain fatty acids is not subject to the regulatory constraints imposed on the oxidation of long‐chain fatty acids. The hepatic production of acetate leads to a significant elevation in blood acetate concentration (Dettling et al., 2007). Previously, the association of movement disorders, including progressive myoclonus epilepsy, ataxia and dystonia, with hepatic disease was reported. It was emphasized that this condition manifests in three primary scenarios: (i) simultaneous involvement of both organ systems due to a single disease entity, (ii) nervous system dysfunction arising from exposure to toxic compounds in the presence of impaired hepatic clearance or (iii) hepatic and/or neurological injury as a consequence of exposure to external drugs or toxins (Mulroy et al., 2021). Thus, higher serum ethanol and acetate concentrations detected in the dogs with tick paralysis than the healthy ones may be associated with reduced liver oxidation capacity and hepatic clearance.

l‐carnitine, which is essential for free fat acid metabolism in mitochondria, was reported to reduce fatigue and improve endurance in patients with muscle wasting due to various conditions (Cuhls et al., 2017). Moreover, it was reported that l‐carnitine maintained mitochondrial function in spinal cord injured rats. Thus, considering the neuroprotection properties of carnitine, the high l‐carnitine level detected in dogs with tick paralysis may be related to hindlimb locomotion sparing and the response to damage (Patel et al., 2012).

Creatine, a nitrogenous organic compound formed through reactions involving the amino acids arginine, glycine and methionine, plays a crucial role in the resynthesis of ATP, especially during periods of heightened metabolic demand, such as sleep deprivation, mental health conditions or neurological diseases (Roschel et al., 2021). Although the vast majority of creatine is synthesized in the kidneys and liver, creatine can also be endogenously synthesized in the brain (Fernandes‐Pires & Braissant, 2022). Moreover, creatine exhibits a certain capacity to traverse the blood–brain barrier through microcapillary endothelial cells expressing the creatine transporter SLC6A8, enabling its accumulation in the brain. Nonetheless, the uptake of creatine in the brain is generally constrained compared to other tissues, such as skeletal muscle. This limitation may be attributed to the potentially low permeability of the blood–brain barrier to creatine and/or the absence of SLC6A8 expression in astrocytes (Hultman et al., 1996). Due to limited cerebral energy availability and injury‐induced cerebral blood flow anomalies, energy supply and demand are uncoupled (Giza & Hovda, 2014). Previously, it was reported that glucose demand is increased in paralytic extremities that have undergone muscle atrophy (Graham et al., 2019). For this reason, the high serum creatine concentration of the dogs with tick paralysis of the present study can be interpreted as a response to the body's increased energy needs considering the fact that neurons require a constant supply of ATP for several cellular processes including neurotransmitter exocytosis and synaptic functioning (Forbes et al., 2022).

Citrate is generated in the mitochondria from acetyl‐CoA and oxaloacetate, entering the citric acid cycle (tricarboxylic acid cycle or Krebs cycle). Beyond its role in energy production, citrates play crucial roles in additional actions, such as down‐regulating inflammation and reducing lipid peroxidation (Abdel‐Salam et al., 2014). The elevated citric acid concentrations of the tick paralyzed dogs of the present study may be related to muscle weakness and mitochondrial impairment that develop in tick paralysis (dos Santos et al., 2016). Moreover, as citrate is an endogenous metabolite with complete and rapid metabolism, elevated citrate concentrations may increase muscle weakness and flaccidity due to its calcium chelating properties (Icard et al., 2021). Therefore, monitoring citrate concentration may have potential use in predicting the prognosis of tick paralysis.

In cases of AFP, the occurrence of renal tubular acidosis was reported (Vandal et al., 2020). Laboratory interpretation in the routine of care is extremely important in order to assist in the detection of alterations of the organism to the pathology. Parameters, such as creatinine and urea, are indicated for the evaluation of renal function. Therefore, in the present study, elevated concentrations of urea of the tick paralyzed dogs could be related to the disruption of the renal function (Santos et al., 2020).

Hypoxanthine is one of the intermediates in the degradation of energy‐rich adenine nucleotides to uric acid and is presumed to accumulate when energy states are reduced by hypoxia, ischemia or academia (Saugstad, 1975). Recently, it has been reported that hypoxanthine infusion in striatum of young Wistar rats increases neuroinflammatory parameters perhaps through oxidative misbalance (Biasibetti et al., 2017). It has been hypothesized that the excess hypoxanthine accumulating as a consequence of hypoxanthine guanine phosphoribosyl transferase deficiency triggers the neurological abnormalities (Torres et al., 2016). The high serum hypoxanthine concentrations of the tick paralyzed dogs of the present study may be related to hypoxia associated with respiratory distress, acidemia due to decreased renal function and neuroinflammation. Given that the cause of neuroinflammation in botulism, another factor associated with AFP, is the sensitivity of dopaminergic pathways to inflammation, one can speculate that a similar neuroinflammatory response may develop in cases of tick paralysis. The evaluation of this hypothesis can be conducted through hypoxanthine assessment, as suggested previously (Ham et al., 2022). Still, further studies are needed.

Formic acid is a standard intermediate in human metabolism, contributing to the processing of one‐carbon compounds, with its carbon potentially appearing in methyl groups during transmethylation. It is commonly generated as a by‐product in acetate production and subsequently oxidized to carbon dioxide. Numerous studies have highlighted the potential harm of formic acid to organs and tissues, with elevated concentrations posing a risk of fatality (Fox & Stover, 2008). Formic acid exerts direct toxic effects, inducing oxidative stress, mitochondrial damage and heightened lipid peroxidation, all contributing to its neurotoxic mechanism. Notably, formic acid inhibits cytochrome c oxidase activity, precipitating severe metabolic acidosis. As a neurotoxic substance, formic acid and its metabolites are associated with permanent blindness, particularly due to their affinity for the optic nerve. Additionally, formic acid induces depression in the central nervous system, systemic metabolic acidosis, and may progress to coma and eventual death (Moral et al., 2015). In the present study, formic acid, which is probably released as a result of impaired energy metabolism due to mitochondrial damage, can be used to predict eye‐related damage and further central nervous system depression in dogs with tick paralysis.

Intact white adipose tissue (WAT), along with isolated adipocytes, actively secretes substantial amounts of glycerol. The conventional understanding has been that this glycerol is a by‐product of lipolysis, released through the action of cell lipases on triacylglycerol (TAG) stores and/or lipoprotein‐carried TAGs, that is, through the activity of lipoprotein lipase (Langin, 2006). WAT capacity to recycle free glycerol is limited, but glycerol is a main substrate for hepatic gluconeogenesis and a viable substrate for energy or TAG synthesis in many tissues (Robinson & Newsholme, 1967). Moreover, glycerol is a potent osmotic dehydrating agent with additional effects on brain metabolism. Moreover, glycerol helps in neural recovery and sustained resolution of cerebral oedema (Shankar, 2019). It was suggested that glycerol dehydrated the nerve fibres sufficiently to decrease their activity and hence hinder the paroxysmal discharges that constitute attacks of trigeminal neuralgia (Fromm et al., 1984). Hence, the elevated serum glycerol levels observed in the dogs affected by tick paralysis could be linked to increased lipolysis, given that dysregulation of lipid metabolism has been implicated in various neurological disorders, although the precise regulatory mechanisms remain unclear (Lee et al., 2022).

Betaine, a stable and nontoxic natural substance ubiquitous in animals, plants and microorganisms, is endogenously synthesized through choline metabolism or obtained through dietary intake. Its primary functions include serving as an osmolyte and acting as a methyl‐group donor (Zhou et al., 2016). Various studies indicate that betaine plays a protective role against alcohol‐induced hepatic steatosis, apoptosis and the accumulation of damaged proteins. Furthermore, it demonstrates a significant ability to prevent or attenuate progressive liver injury by preserving intestinal integrity and adipose function. These protective effects are predominantly linked to the regulation of methionine metabolism, involving the removal of homocysteine and the maintenance of cellular SAM:SAH ratios (Domitrović & Potočnjak, 2016). In addition, betaine has a neuroprotective role, preserves myocardial function, and prevents pancreatic steatosis. Betaine also attenuates oxidant stress, endoplasmic reticulum stress, inflammation and cancer development (Craig, 2004). The elevated concentrations of choline and betaine observed in dogs affected by tick paralysis in this study could be ascribed to liver disease triggered by paralysis, apoptosis, compromised myocardial function and the destruction of adipose tissue as a response. This aligns with the understanding that tick paralysis has the potential to lead to multiple organ failure (Gülersoy & Günal, 2022).

A progressive loss of muscle and collagen has been reported in tetraplegic patients, and the effectiveness of the exposed amino acid chains in the treatment and prediction of the course of the disease has been investigated (Claus‐Walker & Rodriguez, 1980). Additionally, in dogs with hepatic encephalopathy and severe cirrhosis, increased plasma aromatic amino acids such as tyrosine, phenylalanine, methionine and free tryptophan and a decrease in the branched chain amino acids, valine, leucine and isoleucine have been reported. Tryptophan, tyrosine and phenylalanine serve as precursors for physiological biogenic amine neurotransmitters, but they can also give rise to other compounds that may impact normal neurotransmitter metabolism. The elevation of aromatic amine precursors in the brain corresponds to significant alterations in brain amine content, such as decreased norepinephrine levels and increased serotonin levels (Cummings et al., 1976). The detected high levels of amino acids may be associated with the fact that the brain still maintains energy balance, protein synthesis or perhaps even lipid synthesis in cases of tick paralysis.

The essential amino acid l‐tryptophan is acquired through dietary intake and is essential for the synthesis of proteins in every living cell. The metabolism of tryptophan produces various biologically active molecules, including several crucial neuromodulators that play a significant role in central nervous system function. Tryptophan is the only amino acid to bind plasma albumin and exists in an equilibrium between albumin‐bound and free forms in the peripheral circulation (McMenamy, 1965). The low tryptophan level detected may be associated with the development of hypoalbuminemia due to hepatic dysfunction in dogs with tick paralysis.

Glucose undergoes glycolysis in the cytoplasm, converting into pyruvate. Subsequently, pyruvate is oxidized in the mitochondria to produce ATP through the tricarboxylic acid cycle and oxidative phosphorylation. In extended fasting periods, hepatic glycogen is depleted, prompting hepatocytes to engage in gluconeogenesis. This process utilizes precursors such as lactate, pyruvate, glycerol and amino acids for the synthesis of glucose (Burgess et al., 2004). Moreover, it was reported that elevated blood glucose and C‐reactive protein are usually related to a worsened clinical outcome in neurological diseases. The high pyruvate and glucose levels of the dogs with tick paralysis in the present study may be related to stress hyperglycaemia due to impaired glucose homeostasis (Štětkářová et al., 2022).

The most common misdiagnosis in tick paralysis cases has been reported as Guillain–Barré syndrome (Simon et al., 2023). In an untargeted NMR‐based metabolomics study, metabolites with dysregulated expression (such as acetone, creatine, methionine and isoleucine) were identified among variants of Guillain–Barré syndrome including acute inflammatory demyelinating polyneuropathy, acute motor axonal neuropathy and Miller Fisher syndrome (Park et al., 2019), similar to those in the present study (Table 2). Since targeted studies in metabolomics focus on the quantitative concentrations of a small number of metabolites and untargeted studies focus on the metabolic profile of all metabolites in a sample (Griffin, 2004), the untargeted metabolomic analysis method used in this study could not demonstrate that the metabolites with altered expression were specific to tick paralysis. However, the research provided insights into the impaired metabolic pathways in the affected dogs. A comparative metabolomic study of AFP cases with infectious and noninfectious aetiologies, such as tick paralysis, may help demonstrate the specificity of the metabolomic findings. Therefore, further comparative studies with various AFP aetiologies are recommended. Moreover, the absence of principal component analysis, orthogonal projections to latent structures analysis, metabolite network mapping and related pathway analysis for modelling spectroscopic data in the present study can be considered a limitation.

## 5 CONCLUSION

Tick saliva blocks the release of acetylcholine at the neuromuscular junction and clinical signs develop 7–9 days after attachment and engorgement of the tick. If definitive diagnosis is delayed mortality increases. Thus, the lack of methods to be used in definitive diagnosis of tick paralysis can be filled by investigation of the metabolic pathways of the affected host. NMR is a preferred approach in the field of clinical metabolomics due to its capacity to detect all organic molecules as well as its adaptability in the analysis of biological samples. In the present study, NMR‐based serum metabolomics analysis revealed diverse up/down‐regulated expressions, presenting a promising avenue. In addition, it was determined that not only the respiratory system is affected in cases of tick paralysis, but also liver and kidney functions and energy homeostasis are impaired. Among the metabolites whose expression was changed, l‐glutamic acid, acetoacetate, isopropanol, creatine, tyrosine, phenylalanine, methionine and tryptophan can be considered in the evaluation of energy metabolism disruption caused by long‐term starvation due to flaccid paralysis. Ethanol and acetate may be considered in the evaluation of liver oxidation capacity, clearance and function. Moreover, it was observed that neuroinflammation may also develop in cases of tick paralysis and that the neuroprotective response level of the host's body can be examined by evaluating the levels of l‐carnitine, citrate, hypoxanthine, formic acid and betaine. As a result, it was concluded that the present approach may help to better understand the pathological mechanisms developing in tick paralysis. In addition, metabolites with altered expression can be used for diagnostic and/or prognostic purposes in subsequent studies not only in tick paralysis but also in other AFP cases.

## AUTHOR CONTRIBUTIONS

**Erdem Gülersoy**: Writing– original draft writing–review and editing; investigation; data curation; software; formal analysis; conceptualization; resources; project administration. **Canberk Balıkçı**: Writing–review and editing; validation; software; formal analysis; investigation; data curation. **Adem Şahan and İsmail Günal**: Writing–original draft; visualization. **Mehmet Osman Atlı**: Writing–review and editing; visualization; resources.

## CONFLICT OF INTEREST STATEMENT

The authors declare that they have no conflicts of interest or personal relationships that could have appeared to influence the work reported in this paper.

## ETHICS STATEMENT

This study was approved by the Harran University Animal Experiments Local Ethics Committee (Date: 09.05.2022, Number: 2022/003 – 01/06 Ethics Committee Decision). Moreover, all dog owners gave their consent before the commencement the study. No experimental practices that will or can harm the animals or put their welfare at risk were done.

### PEER REVIEW

The peer review history for this paper is available at https://publons.com/publon/10.1002/vms3.1528.

## Data Availability

The data that support the study findings are available from the authors upon request.
